# Copper, zinc and iron levels in infants and their mothers during the first year of life: a prospective study

**DOI:** 10.1186/s12887-015-0474-9

**Published:** 2015-10-14

**Authors:** Tülin Ayşe Özden, Gülbin Gökçay, M. Serdar Cantez, Özlem Durmaz, Halim İşsever, Beyhan Ömer, Günay Saner

**Affiliations:** Department of Pediatrics, Istanbul Faculty of Medicine, Istanbul University, Trace Element Unit, 34093 Istanbul, Turkey; Institute of Child Health and Istanbul School of Medicine Department of Pediatrics, Istanbul University, 34093 Istanbul, Turkey; Department of Pediatric Gastroenterology, Istanbul School of Medicine, Istanbul University, 34093 Istanbul, Turkey; Department of Public Health, Istanbul School of Medicine, Istanbul University, 34093 Istanbul, Turkey; Department of Biochemistry, Istanbul School of Medicine, Istanbul University, 34093 Istanbul, Turkey

**Keywords:** Infant, Mother, Serum trace elements, Hair trace elements, Breastfeeding, Diet

## Abstract

**Background:**

Essential micronutrients are important for maintenance of life. Deficiency of micronutrients is more likely to be encountered in children, and women studies are required to investigate the status of micronutrients in children and women. This study aimed to longitudinally evaluate changes in zinc, copper, and iron levels in breastfed infants and their mothers during the first year of life.

**Methods:**

Serum and hair samples were obtained from 35 healthy breastfed infants (51 % males, 49 % females) and their mothers 2, 6, and 12 months after delivery. All of the samples were assessed using an atomic absorption spectrophotometer. Serum iron levels were determined by a Roche/Hitachi/Modular analyzer. Statistical analyses were performed using SPSS-PC (Version 21.00) software.

**Results:**

Hair zinc (*p* < 0.05) and serum iron (*p* < 0.001) levels of infants were significantly decreased towards the end of the first year. Infants’ serum copper levels were increased towards the end of the first year. Maternal serum and hair copper levels and serum iron levels were significantly decreased towards the end of the first year. There were no significant correlations between dietary zinc, copper, iron intake, and trace element levels of infants and their mothers.

**Conclusions:**

Infants’ hair zinc levels, maternal and infants’ hair copper levels, and infants’ and maternal serum iron levels declined towards the end of the first year. Infants need more zinc after 6 months of age. Infants’ and mothers’ daily iron intake was less than the recommended intake.

## Background

Copper (Cu), zinc (Zn), and iron (Fe) are essential micronutrients for maintenance of life. These micronutrients are involved in many complex enzyme systems functioning in various biological processes [1–6]. Deficiency of trace element nutrients is more likely to be encountered in children, and pregnant and lactating women [7, 8]. There are interactions between some trace elements. Deficiency in one trace element may impair absorption of another (e.g., Cu deficiency impairs Fe absorption). Fe and Zn interact at the level of the intestinal mucosa and Zn absorption is impaired by Fe [9, 10]. There is also a strong interaction between Zn and Cu, and they compete at the level of intestinal absorption [11]. High Zn levels in the diet can reduce the absorption of Cu, but high dietary Cu does not decrease absorption of Zn [12].

Inadequate intake of Zn is considered to be responsible for 20 % of global child mortality [13]. Children with iron deficiency anemia have high serum Cu levels and low serum Zn levels [14]. Trace element deficiencies arise from low dietary intake and develop especially when requirements are increased or body stores are depleted. Absorption of trace elements may be impaired by increased intake of dietary components, such as phytate or excessive intake of mineral supplements [11, 15]. Another possible mechanism for trace element deficiency is excessive excretion or use. Zinc and copper deficiency is also found in malabsorption syndromes, such as chronic diarrhea, coeliac disease, inflammatory bowel disease, ileostomy, alcoholic cirrhosis, and hemolytic anemia [16].

Zinc as a trace element has three important functional roles: catalytic, structural, and regulatory [3, 5]. Copper has an antioxidant role that protects cells from free-radical injury [3, 17]. Copper also contributes to the formation of ceruloplasmin, which has a role in iron metabolism. Copper is required to absorb and use Fe [1, 14, 17, 18]. Infants and young children in developing countries are particularly vulnerable to Fe and Zn deficiency because of increased requirements, low bioavailability, and recurrent infections [7, 18].

Copper deficiency is rare, but it has been reported in preterm infants, in infants fed with cow’s milk, and in infants recovering from malnutrition accompanied by diarrhea [6, 19–21]. Deficiency of Cu leads to anemia, neutropenia, impairment of growth, abnormalities in glucose and cholesterol metabolism, and increased rates of infection [22].

Iron is another essential trace element that functions in the synthesis of hemoglobin and myoglobin [23]. A total of 25 % of the world’s population is thought to be affected by Fe deficiency. Infants aged between 4 and 24 months, school-age children, females, adolescents, and pregnant and lactating mothers are most affected by this deficiency [24].

Serum concentrations are useful parameters to assess trace elements, but they are not sufficiently specific and sensitive to detect mild deficiency [25, 26]. Hair shaft Zn and Cu levels are useful parameters to determine the quantity of trace elements that is available to the hair follicles at the time of growth, rather than the actual time that children are sampled. Hair trace element levels have been proposed as a useful index of the long-term status of trace elements [27]. Therefore, studies are required to investigate the status of trace elements and their interactions among each other in infants. To the best of our knowledge, there are no longitudinal cohort studies that have investigated the Cu, Zn, and Fe status of breastfed infants and their mothers. There is one relevant study, but it is not a cohort study [28].

Therefore, the present study aimed to longitudinally evaluate the changes in Zn, Cu, and Fe levels of breastfed infants and their mothers after delivery during the first year of life.

## Methods

This longitudinal study was conducted between December 2007 and January 2010 in two month-old infants who were attending the Well Child Unit of Istanbul Medical School, Istanbul University. Blood and hair samples were obtained from 111 infants and their mothers 2 months after delivery. Although there were 111 infants and mothers at the beginning of the study, we lost 76 participants (loss to follow-up group) because of infection, medicine use, and vitamin use, and some did not continue to visit the clinic. Blood and hair samples were collected longitudinally from 35 infants (18 males, 17 females) and their mothers 2, 6, and 12 months after delivery.

Inclusion criteria for the study were as follows: uneventful pregnancy, term delivery, and birth weight ≥ 2500 g with no apparent congenital defects. Children born at the Maternity Clinic of the University Hospital constituted the majority of the infants and the children who were followed up at the clinic. Specimens were collected by convenience sampling. Children with any proven or suspected infection at the time of collection of samples were excluded from the study. All of the samples were assessed using an atomic absorption spectrophotometer (Varian Spectra AA 200, GTA-100, Australia). Serum Fe levels were determined by a Roche/Hitachi/ Modular analyzer, japan.

A complete physical examination, including anthropometric measurements, was performed for each infant. Weight, length, and head circumference measurements were performed by two trained nurses. Z scores for length, weight, and head circumference measurements of infants were calculated with a computerized program that was developed for Turkish children [29, 30]. This study was supported by the Istanbul University Research Fund. Written consent was obtained from the parents. Approval of the Medical School Ethical Committee was obtained at the beginning of the study.

### Collection of data and specimens, and laboratory procedures

A validated questionnaire that was specific for this project was developed in a pilot study to collect data on the feeding habits of infants and their mothers. All of the mothers were on Fe and folic acid supplementation during pregnancy. Daily and weekly consumption of meat, milk, eggs, and vegetables was recorded. Infants were classified as either exclusively breastfed (infants receiving only breast milk, not even water), partially breastfed, or non-breastfed. Data on daily intake of Zn, Cu, Fe, and meat in infants and their mothers were calculated using a computerized nutrient analysis program (BEBiS), which has been adapted for Turkish infants and their families. Infants’ dietary habits were evaluated only once at 12 months and mothers’ dietary habits were evaluated 2, 6, and 12 months after delivery. For evaluation of breast milk intake, the duration of each feed was used to estimate the likely volume of milk. A feed lasting 10 min or longer was assumed to be 100 ml in volume (i.e., 10 ml per min) and a proportion of this if the feed was of shorter duration [31]. For example for a feeding lasting 5 min, the milk intake was assumed to be 50 ml.

Blood specimens were collected after a fasting period of 8 or 10 h for mothers and 4 h for infants. Trace element-free syringes, stainless steel needles, and special trace element tubes (Becton-Dickinson) were used.

The serum samples were separated after 10 min of centrifugation. Serum samples were diluted at a 1:6 ratio with bi-distilled water. Serum Zn and Cu concentrations were measured using an atomic absorption flame emission spectrophotometer (Varian AA 100, Australia) (213.9 nm and 324.8, respectively) [32, 33]. A standard curve was established using a commercial Zn and Cu reference (Merck KGaA Darmstadt, Germany). The coefficient of variation of the measurements was always below 5 %.

Hair samples of infants and their mothers were collected from the suboccipital area of the head. Divided hair samples were sequentially washed three times in hexane, analytical grade ethanol, and fresh bi-distilled water. They were dried at 75 °C in a vacuum oven overnight in polyethylene vial and weighed 20–100 mg. The hair was digested using perchloric acid and nitric acid. Digestion was performed between 65 and 75 °C [33, 34]. The ashed samples were dissolved in 1 mL of bi-distilled water and 10-μL aliquots were injected into a graphite furnace with an auto sampler. Bovine liver certified standard (SRM no. 1577c certified; National Institute of Standard and Technology) and a pooled hair sample were similarly digested in perchloric acid and nitric acid, and were used as internal standards. A standard curve was established using a commercial Zn and Cu reference (Merck KGaA). Hair Zn and Cu concentrations were determined using a Varian Spectra AA 200 atomic absorption spectrophotometer equipped with a GTA-100 [32–34]. Hair Zn and Cu levels are expressed in μmol/g, and serum levels of Zn and Cu are expressed in μmol/g. Serum and hair Zn levels lower than 10.70 μmol/L and 1.07 μmol/g, respectively, were accepted as low levels [27, 35]. Normal serum Cu levels have been reported as between 3.15 and 11 μmol/L in infants aged up to 6 months of age, (mean ± SD) 17.50 ± 4.10 μmol/L for infants aged from 6 months to 2 years, and 12.60-24.40 μmol/L for females [36–38]. There is no exact cut off level for hair Cu levels for infants in the literature. Serum iron levels less than 8.95 μmol/L were accepted as low [39].

### Statistical analysis

Statistical analyses were performed using SPSS-PC (IBM Corp; Version 21.00) software. Pearsonχ^2^analysis for non-continuous, the Student’s *t*-test for continuous, and the Mann–Whitney *U* test for non-normal distribution variables were performed between the groups of “loss to follow-up” and “completed the study”.

Final analysis of the study was based on the ones completed the study. Data of hair samples did not have a normal distribution. Therefore, *χ*^2^ Friedman and Wilcoxon ranks tests were used for analyses. Data of serum Zn, Cu, and Fe levels had a normal distribution. Therefore, repeated ANOVA and Paired sample t-tests were used for these cohort specimens. One Way ANOVA test was used to compare daily Zn, Cu, and Fe intake according to months. Spearman’s and Pearson’s correlation tests were used to determine relationships. Values of *p* < 0.05 were accepted as statistically significant.

## Results

This study was limited to neonates who were born at the Maternity Clinic of Istanbul Medical School. At discharge from the Maternity Clinic, each mother received a pamphlet with information on the Well Child Clinic. Families had relative socio-economic and cultural homogeneity in this study. All of the families were well above the poverty line, as assessed by their ability to bring their neonate to our center. All of the parents were literate. The majority of the mothers were high school graduates. Preterm infants born before 37 gestational weeks were not followed up at the Well Child Clinic.

The majority of the parents in the study had at least 5 years of schooling. Sociodemographic characteristics of infants and their mothers are shown in Table 1. Weight, length, and head circumference Z scores of all of the infants were within normal limits (Table 2). All 35 infants (18 males, 17 females) and their mothers were followed up until the children were aged 1 year. The breastfeeding status of all infants, parity, and maternal age are shown in Table 1. Hair trace element levels were not normally distributed. Therefore, 95 % confidence intervals and median levels are shown in Table 3.

**Table 1:** Comparison of sociodemographic characteristics of infants and their families

Variables	Loss to follow-up		Completed the study		
	*n* = 76	%	*n* = 35	%	Two-tailed significance
Sex					
Male	42	55.30	18	51.50	^a^0.14; *p* > 0.05
Female	34	44.70	17	48.50	
Maternal education					
Primary school	30	39.40	8	23	^a^2.95; *p* > 0.05
Completed high school	25	33.00	15	43	
University graduate	21	27.60	12	34	
Paternal education					
Primary school	20	26.30	5	14.40	^a^3.31; *p* > 0.05
Completed high school	28	36.80	12	34.30	
University graduate	28	36.80	18	51.30	
Maternal occupation					
Employee	11	14.47	3	8.60	^a^0.63; *p* > 0.05
Clerk	27	35.53	13	37.20	
^c^Physician	1	1.32	2	5.60	
Housewife	35	46.05	16	45.70	
^c^Others	2	2.63	1	2.90	
Feeding status of infants at 2 months					
Exclusively breastfed	63	83.00	31	88.60	^a^0.59; *p* > 0.05
Breast milk + other food	13	17.00	4	11.40	
Feeding status of infants at 6 months					
Exclusively breastfed			13	37.10	
Breast milk + other food			22	62.90	
Feeding status of infants at 12 months					
Breast milk + other food			35	100	
				Mean ± SD	
Maternal age (years)	76	30.12 ± 4.95	35	31.30 ± 4.50	^b^t = 1.20; *p* > 0.05
Parity	76	1.70 ± 0.66	35	1.80 ± 0.90	^b^t = 0.5; *p* > 0.05

^a^Pearson χ^2^ analysis and ^b^ Student’s t-tests were performed

^c^Physicians and others were excluded from the statistical analysis because of their small numbers

**Table 2:** Z scores of the infants’ length, weight, and head circumference

Months	2	6	12
Length			
Mean ± SD; median 95 % CI	0.18 ± 0.91; 0.04 (−0.13)-(0.48)	0.36 ± 0.93;0.14 (0.052)-(0.67)	0.56 ± 0.92;0.34 (0.26)-(0.86)
Weight			
Mean ± SD; median 95%CI	0.22 ± 1.07; 0.15 (−0.13)-(0.57)	0.22 ± 0.96; 0.46 (−0.1)-(0.54)	0.10 ± 0.94;0.12 (−0.21)-(0.41)
Head circumference			
Mean ± SD; median 95 % CI	−0.33 ± 1.08;-0.50 (−0.68)-(0.03)	−0.35 ± 0.88;-0.68 (−0.64)-(−0.06)	−0.36 ± 0.89; −0.62 (−0.65)-(−0.07)

Values are mean ± SD, median levels, and 95 % CIs. *CI* confidence interval

**Table 3:** Infants’ and mothers’ serum Zn, Cu, and Fe, and hair Zn and Cu levels

	Maternal (*n* = 35)			Total (*n* = 105)	Infants (*n* = 35)			Total (*n* = 105)
Months after delivery	2	6	12		2	6	12	
Serum Zn (μmol/L)^a^	15.00 ± 3.10^c^	14.80 ± 1.50^c^	14.10 ± 3.10^c^	14.7 ± 2.75^c^	13.30 ± 1.50^d^	13.60 ± 3.10^d^	13.50 ± 3.10 ^d^	13.30 ± 2.70 ^d^
Serum Cu (μmol/L)^a^	19.70 ± 4.70^e^	18.70 ± 3.10^e^	18,3 ± 3.30^e^	19 ± 3.3^e^	14.6 ± 3.10^f^	17.20 ± 3.30^f^	18.00 ± 3.10^f^	16.50 ± 3.10^f^
Serum Fe (μmol/L)^a^	13.10 ± 5.6^g^	13.71 ± 5.40^g^	12.10 ± 5.60^g^	13 ± 3.8^g^	12.4 ± 3.80^h^	8.70 ± 3.20^h^	8.5. ± 3.70^h^	9.85 ± 4.10^h^
Hair Zn (μmol/g)^b^ (median; 95 % CI lower-upper level)	1.48 ± 0.67^i^ (1.50;1.28–1.72)	1.84 ± 0.75^i^ (1.65;1.60–2.10)	1.76 ± 0.80^i^ (1.54;1.50–20)	1.70 ± 0.74^i^ (1.50;1.56–1.84)	1.30 ± 0.73^j^ (1.27; 1.06–1.55)	1.02 ± 0.50^j^ (0.86; 0.87–1.18)	0.77 ± 0.30^j^ (0.79;0.67–0.86)	1.03 ± 0.60^j^ (0.88;0.92–1.14)
Hair Cu (μmol/g)^b^ (median; 95 % CI lower-upper level)	0.20 ± 0.11^k^ (0.17;0.16–0.24)	0.22 ± 0.11^k^ (0.19; 0.18–0.26)	0.17 ± 0.19^k^ (0.15;0.11–0.19)	0.20 ± 0.11^k^ (0.17;0.18–0.22)	0.32 ± 0.14^l^ (0.32;0.24–0.34)	0.34 ± 0.16^l^ (0.32;0.28–0.39)	0.25 ± 0.13^l^ (0.25;0.22–0.32)	0.30 ± 0.16^l^ (0.25;0.27–0.33)

Values are mean ± SD unless stated otherwise

^a^Repeated ANOVA and the paired sample *t*-test were used to compare serum Zn, Cu, and Fe levels

^b^χ^2^ Friedmanand Wilcoxon rank tests were used for hair analysis

^c^F_2,6_ = 0.66, *p* > 0.05; F_2,12_ = 2.42, *p* > 0.05, t _6,12_: 1,24, *p* > 0.05

^d^F_2,6_ = 0.70, *p* > 0.05; F_2,12_ = 0.61, *p* > 0.05, t _6,12_: 0.09, *p* > 0.05

^e^F_2,6_ = 2.07, *p* > 0.05; F_2,12=_3.65, *p* > 0.05;t _6,12_: 0.99, *p* > 0.05

^f^F_2,6_ = 8.65, *p* < 0.01; F_2,12=_28.03, *p* > 0.001;t _6,12_: 1,29, *p* > 0.05

^g^F_2,6_ = 0.47, *p* > 0.05; F_2,12=_0.64, *p* > 0.05;t _6,12_: 1,28, *p* > 0.05

^h^F_2,6_ = 21.9, *p* < 0.001; F_2,12=_17.35, *p* < 0.001;t _6,12_: 0.36, *p* > 0.05

^i^χ ^2^_Friedman_ = 9.77, *p* < 0.05; Z_2,6_ = 2.57, *p* < 0.05, Z_2,12_ = 2.58, *p* < 0.05

^j^ χ^2^_Friedman_ = 19.94, *p* < 0.001; Z_2,6_ = 2.06, *p* < 0.05, Z_6,12_ = 2.53, *p* < 0.01, Z_2,12_ = 4.09, *p* < 0.001

^k^χ ^2^_Friedman_ = 10.7, *p* < 0.005; Z_2,6_ = 2.12, *p* < 0.05, Z _2,12_ = 2.24, *p* < 0.05, Z _6,12_ = 3.00, *p* < 0.005

^l^χ ^2^_Friedman_ = 8,56, *p* < 0.05; Z_6,12_ = 3.15, *p* < 0.05, Z _2,12_ = 2351, *p* < 0.5

With regard to sociodemographic characteristics, there were no significant differences between the groups “loss to follow-up” and “completed the study” (Table 1). There were no differences in trace elements between these groups.

Infants’ and mothers’ serum zinc levels were not significantly different during the follow-up period (Table 3). As shown in Table 3 hair Zn levels of infants were significantly lower at the ages of 6, 12 months than those at 2 months (*p* < 0.05; *p* < 0.001, respectively). Mothers’ hair zinc levels were significantly higher at 6 months after delivery compared with those at 2 and 12 months (Table 3). Three (8.50 %) infants in the 2nd month, five (14.30 %) in the 6th month, and 6 (17 %) at 1 year had serum Zn levels below 10.70 μmol/L. Among these infants, 48.60, 66 and 77.10 % had hair Zn levels below 1.07 μmol/g at 2, 6, and 12 months, respectively. Among the mothers, 14.30, 2.90, and 2.90 % had serum Zn levels below 10.70 μmol/L, and 20, 11.40, and 11.40 % had hair Zn levels below 1.07 μmol/g at 2, 6, and 12 months after delivery, respectively (Table 4).

**Table 4:** Zinc and iron status of mothers and infants after delivery

Months after delivery		Serum Zn < 10.70 μmol/L		Hair Zn < 1.07 μmol/g		Serum Fe < 8.90 μmol/L	
		n	%	n	%	n	%
Mothers (*n* = 35)	2	5	14.30	7	20	8	23
	6	1	2.90	4	11.40	4	11.40
	12	1	2.90	4	11.40	10	28.60
Infants (*n* = 35)	2	3	8.50	17	48.60	11	31.40
	6	5	14.30	23	66.00	18	51.40
	12	6	17.00	27	77.10	22	62.90

Infants’ serum Cu levels at 12 months of age were significantly higher those at 2 and 6 months (Table 3). Infants’ serum Cu levels in the total group (*n* = 105) ranged between 8.70 and 26.80 μmol/L. Hair Cu levels were a minimum of 0.21 μmol/g and a maximum of 0.35 μmol/g in infants and a minimum of 0.14 μmol/g and a maximum of 0.26 μmol/g in mothers. Hair Cu levels were not normally distributed. Therefore, 95 % confidence intervals and median levels are shown in Table 3. Infants’ and mothers’ hair Cu levels were significantly higher at 6 months compared with 2 and 12 months after delivery (Table 3). Maternal serum and hair Cu levels at 12 months were significantly lower than those at 2 and 12 months. Serum Cu levels of mothers in the total group (*n* = 105) varied between 13.4 and 24.20 μmol/L.

Serum Fe levels of the infants were significantly lower at 12 months than those at 2 and 6 months (Table 3). Maternal serum Fe levels reached a maximum level at 6 months, and then were significantly decreased at 12 months (Table 3). Among the infants, 31.40, 51.40, and 63 % had serum Fe levels less than 8.95 μmol/L at 2, 6, and 12 months, respectively. Among the mothers, 23, 11.40, and 28.60 % had serum Fe levels less than 8.95 μmol/L at 2.6 and 12 months, respectively (Table 4).

The mean daily Zn, Cu, and Fe intakes of infants aged 12 months were 3.2 ± 1.2 mg, 0.79 ± 0.32 mg, and 3.71 ± 1.43 mg, respectively (Table 5). The mean daily Zn, Cu, and Fe intakes for mothers were 8.20 ± 2.80 mg, 1.38 ± 0.62 mg, and 9.15 ± 2.90 mg, respectively. In the second month, the mothers’ daily Zn, Cu, and Fe intakes were higher than those at 6 and 12 months (*p* < 0.005) (Table 5). Among the mothers, 7.40, 59.20, and 8.50 % consumed red meat, vegetables, and fruit, respectively, every day, but 70.60 % consumed meat less than 2 days a week. There were no significant relationships between dietary Zn, Cu, and Fe intake and the status of trace elements of infants and their mothers.

**Table 5:** Daily trace element intake of mothers and infants after delivery

Months	Mothers					
	Zn		Cu		Fe	
2	9.7 ± 3.80	F = 6,63; *p* < 0.005	1.69 ± 0.56	F = 6,58; *p* < 0.005	10.80 ± 3.25	F = 8.26; *p* < 0.005
6	7.4 ± 2.50		1.24 ± 0.59		8.30 ± 3.10	
12	7.6 ± 2.20		1.20 ± 0.72		8.40 ± 2.30	
Total	8.2 ± 2.80		1.38 ± 0.62		9.15 ± 2.90	
Infants						
12	3.20 ± 1.20		0.79 ± 0.32		3.71 ± 1.43	

Values are mean ± SD

One Way ANOVA test was used to compare daily Zn, Cu, and Fe intake according to months

Significant positive and negative correlations between trace elements in mothers and infants are shown in Table 6.

**Table 6:** Significant correlations among trace elements in hair and serum

Variants	M serum Zn (month 2)	M hair Zn (month 6)	M serum Cu (month 2)	M serum Cu (month 6)	M serum Cu (month 12)	M hair Cu (month 2)	M hair Cu (month 6)	M hair Cu (month 12)	I serum Fe (month 6)	I serum Fe (month 12)	I hair Zn (month 12)
M hair Zn at 2 months	*-0.48	*0.55				*-0.36					
	**0.01	**0.001				**0.05					
M hair Zn at 6 months							*-0.42				
							**0.01				
M serum Fe at 2 months			*-0,40								
			**0.05								
M serum Fe at 6 months									*0.38		
									**0.03		
M serum Fe at 12 months										*0.44	
										**0.04	
M serum Cu at 2 months				*0.62	*0.41						
				**0.001	**0.01						
M serum Cu at 6 months					*0.49						
					**0.01						
M hair Cu at 2 months							*0.78	*0.47			
							**0.01	**0.00			
M hair Cu at 6 months								*0.41			
								**0.01			
I serum Zn at 6 months									*-0.42		
									**0.01		
I hair Zn at 6 months											*0.51
											**0.00

Pearson’s correlation analysis was used for serum

Spearman’s correlation analysis was used for hair

*M* mothers, *I* infants; *r value; **p < value

## Discussion

This study is one of the few longitudinal studies regarding the status of trace elements in predominantly breastfed healthy infants and their mothers. We found that hair zinc and serum iron levels of infants were significantly lower, while serum copper levels were higher at 12 months than those at 2 and 6 months. Maternal serum and hair copper levels and serum iron levels were significantly decreased in the same period. Zinc, copper, and iron are the predominant nutritional trace elements [7, 8, 40]. The regulatory mechanisms of Zn, Cu, and Fe homeostasis are different during pregnancy, lactation, and infancy [8, 18, 41, 42]. Further studies are needed to investigate this issue.

### Zinc

During infancy and early childhood, hair zinc concentrations decline from high neonatal values to minimum values at approximately 2–3 years [27]. This trend in hair Zn concentrations may arise from gradual depletion of tissue Zn pools induced by rapid growth. The International Zinc Nutrition Consultative Group concluded that breast milk is a sufficient source of zinc for normal birth weight term infants until approximately 6 months of age [27, 43–45]. Changes in hair Zn concentrations of breastfed and bottle-fed infants during the first 6 months of life were measured by MacDonald et al. [46], and only the bottle-fed males had a significant decline in hair Zn concentrations. There was no decline in hair Zn concentrations in any breastfed infants [46]. In our study, infants’ hair Zn levels significantly declined from high levels at 2 months to low levels at 6 and 12 months (Table 3). Children start to lose endogenous zinc from non-intestinal sites, such as the urinary tract and skin, after 6 months of age, because infants need more Zn after 6 months of age [6, 27]. All of the infants’ eating habits were included in this evaluation. The infants’ dietary habits for Zn, Cu, and Fe were evaluated only at 12 months using a computerized nutrient analysis program (BEBiS), which has been adapted for Turkish infants and their families.

A meta-analysis in Turkey that included 17 studies showed that the mean serum Zn level of 336 children aged between 1 and 3 months was 14.10 ± 3.70 μmol/L [47]. Another meta-analysis of 28 studies in Turkish adults (*n* = 4298) showed that the mean serum Zn level was 14.50 ± 2.91 μmol/L [47]. In our study, the mean serum Zn level of infants at all ages was slightly lower than that in previous results mentioned above [47]. However, the mothers’ serum Zn levels were similar to those of Turkish adult levels.

Some authors have reported that dietary maternal zinc intake during lactation is approximately 13–15 mg/day [44, 48]. The recommended intake of Zn for lactating mothers is 12–13 mg /day [49]. The mean daily dietary zinc intake of lactating mothers in our study was lower than these estimated requirements. Similar low findings have also been previously reported for lactating mothers [50, 51]. In our study, the mean daily Zn intake of all infants at 12 months was close to the recommended intake (3 mg/day) for infants aged 7–12 months [6]. Nevertheless, we did not gather information on phytate-containing food intake in our study.

### Copper

There are few studies on children’s hair Cu levels [52, 53]. Park et al. [52] reported that the mean hair copper level in 655 preschool children was 0.24 ± 0.16 μmol/g. Throughout the whole study period hair Cu levels changed between 0.27 and 0.33 μmol/g in infants and between 0.18 and 0.22 μmol/g in mothers. These results are similar to those in the [52–55]. Eatough et al. [54] reported that hair Cu levels slightly decreased with age. Maternal and infants’ hair Cu levels reached their maximum level at 6 months and then decreased at 1 year in our study. Salmenpera et al. [55] reported that infants’ serum Cu levels increase with age and reach adult levels by 6 months of age. In the current study, serum Cu levels in infants in the 2nd month were lower than those at 6 and 12 months. Serum Cu levels of infants in the total group varied between 8.60 and 26.70 μmol/g throughout the study period. In our study, changes in serum Cu level in infants were similar to those previously reported [5, 14, 55]. Infants’ serum Cu levels at 12 months were higher; whereas maternal serum Cu levels were lower those at 2 and 6 months. There were positive correlations between maternal serum and hair Cu levels at all time periods (Table 6). These correlations showed that factors that affect the maternal Cu status after delivery did not change during the first year. All Cu analyses were performed using an atomic absorption spectrophotometer. We believe that there was minimum measurement error in our study.

The mean daily Cu intake of all infants at 12 months of age was 0.79 ± 0.32 mg in our study. The mean daily Cu intake among all mothers at all time periods was 1.38 ± 0.62 mg. These values are close to the recommended intake for infants and mothers. The estimated safe and adequate daily Cu dietary intake recommended by the Food and Nutrition Board for adults is 1.50-3.00 mg/day [56]. The average Cu intake of children is 0.80–1.90 mg/day [11]. Children (0–0.50 years) often have a low intake of Cu (0.08–0.16 mg/day) because of low Cu levels in breast milk [11]. Despite the declining Cu levels in breast milk during lactation, serum Cu levels in infants are increased, which suggests that the Cu requirements of infants are met. Cu in breast milk appears to be well absorbed and copper levels in breast milk are independent of maternal status [11, 55–57]. Salmenperaet al. [55] showed that serum Cu levels were not correlated with daily Cu intake in infants and in mothers. In our study, there were no relationships between daily intake of Cu and serum and hair Cu levels in infants and mothers. These findings suggest that Cu status is affected by multiple factors other than dietary intake.

The serum Cu level is a good indicator of Cu deficiency. However, neither serum Zn nor Cu reflects marginal status [26]. Therefore, hair Cu levels have been used as an indicator of copper status, particularly in infants [53, 58]. There were negative correlations between maternal hair Cu and Zn levels at 2 and 6 months after delivery in our study (Table 6).

### Iron

Infants, children, and women during fertile years are particularly prone to Fe deficiency. In children, the highest prevalence of Fe deficiency is found between 4 months and 3 years of age because of rapid growth and inadequate dietary intake of Fe [9]. In our study, serum Fe levels decreased with age in mothers and infants. Infection was excluded in the subjects by their history, a physical examination, and complete blood count, which were performed at each clinic visit. We did not measure C-reactive protein levels, which may be a limitation of our study. Infants’ and mothers’ daily Fe intakes were less than the recommended intake [48]. There were positive correlations between infants’ and mothers’ serum Fe levels at 6 and 12 months (Table 6). This finding suggests that dietary Fe intake should be supplemented for mothers and infants. We did not evaluate Fe deficiency anemia and Fe deficiency. We only evaluated elemental Fe status and intake in mothers and infants after delivery for up to 1 year. Although ferritin and transferrin receptor need to be determined for Fe status, we only evaluated trace element levels in the study participants.

There is antagonism among Zn, Cu, and Fe absorption from the gastrointestinal tract. Increased Fe concentrations in the intestinal lumen may block the uptake of Zn [14, 15]. Copper plays a role in Fe metabolism through ceruloplasmin [4]. Dietary Zn absorption is inhibited by Fe [15]. Infants’ hair Zn levels and maternal and infants’ hair Cu and serum Fe levels declined towards the end of the first year. Our study consisted of healthy children. We found a significant negative correlation between the infants’ serum Fe and Zn levels at 6 months (Table 6). Voskaki et al. [3] reported significant correlations between serum Zn and Cu levels in children aged 13–14 years and their mothers. We did not find such a correlation in our study group. The reason for this discrepancy between studies may be due to our small sample size and different age.

Our study is one of the few studies on trace element levels of healthy breastfed infants and their mothers. Nevertheless, our study has some limitations. The families were generally from the middle socio-economic class and were not representative for all of the country. Evaluation of 3-day diets was based on the mothers’ reports and our sample size was small. Therefore, further research is required on a larger scale with participation of families from all socio-economic classes. Additionally, dietary components, such as phytate, which affect Zn, Cu, and Fe metabolism, were not assessed. This is a confounder and could affect absorption of trace elements. We only evaluated elemental Fe status and Fe intake in mothers and infants after delivery for 1 year. Our study aim was not to investigate the mechanism of possible Fe deficiency anemia, but rather to investigate the natural course of Fe levels of breastfed infants and their mothers. However, in further studies, ferritin and transferrin receptor levels should be analyzed to understand the possible mechanisms of Fe deficiency anemia. Although levels of inflammation can affect serum Cu, Fe, and Zn concentration, even if subclinical [59], hair trace element levels are not affected by acute infection [27, 58]. We did not measure C-reactive protein levels, which may have also been a limitation of our study. However, infection was excluded in all subjects by recording the subjects’ history, performing a physical examination, and measuring the complete blood count, at each clinic visit.

## Conclusions

Infants’ hair Zn levels, maternal and infants’ hair Cu levels, and infants’ and maternal serum Fe levels declined towards the end of first year. We observed a significant decline in hair Zn levels of infants at 6 and 12 months than those at 2 months. Children lose endogenous zinc from non-intestinal sites (i.e., urine and body surface) after 6 months of age. Therefore, they need more zinc from that age. Infants’ and mothers’ daily Fe intake was less than the recommended intake. There were positive correlations between infants’ and mothers’ serum Fe levels at 6 and 12 months. This finding suggested that dietary Fe intake should be supplemented for mothers and infants.

## Abbreviations

Zn: Zinc
Cu: Copper
Fe: Iron
